# SNHG26 Promotes Colorectal Cancer Progression via CDKN2A‐Dependent Regulation of Cuproptosis and CD8+ T Cell‐Mediated Immunity

**DOI:** 10.1111/jcmm.70913

**Published:** 2025-11-27

**Authors:** Ziang Wan, Shan Gao

**Affiliations:** ^1^ General Surgery, Cancer Center, Department of Colorectal Surgery Zhejiang Provincial People's Hospital (Affiliated People's Hospital), Hangzhou Medical College Hangzhou Zhejiang China

**Keywords:** CDKN2A, colorectal cancer, cuproptosis, immune evasion, long non‐coding RNA, SNHG26

## Abstract

Long non‐coding RNAs (lncRNAs) play important roles in colorectal cancer (CRC) progression. However, the biological function and regulatory mechanism of small nucleolar RNA host gene 26 (SNHG26) in CRC remain largely unexplored. SNHG26 expression was analysed in CRC tissues and cell lines using quantitative real‐time PCR (qRT‐PCR). The biological functions of SNHG26 were investigated through loss‐ and gain‐of‐function approaches. Interaction between SNHG26 and CDKN2A was examined by RNA immunoprecipitation, RNA pull‐down and RNA stability assays. The effects of the SNHG26‐CDKN2A axis on Cu + ELES(copper plus elesclomol)‐induced cuproptosis and CD8+ T cell‐mediated anti‐tumour immunity were evaluated through cell viability, apoptosis, co‐culture cytotoxicity and migration assays. SNHG26 was significantly upregulated in CRC tissues and cell lines, with high expression showing trends toward poor prognosis. SNHG26 knockdown suppressed CRC cell proliferation and enhanced apoptosis. Additionally, it increased sensitivity to Cu + ELES‐induced cuproptosis. Mechanistically, SNHG26 directly interacted with CDKN2A mRNA, promoting its degradation. CDKN2A, which exhibits context‐dependent effects in CRC, was post‐transcriptionally regulated by SNHG26. Rescue experiments demonstrated that CDKN2A knockdown partially reversed the oncogenic effects of SNHG26 overexpression, including enhanced proliferation, reduced apoptosis and increased resistance to cuproptosis. Furthermore, the SNHG26‐CDKN2A axis modulated the tumour immune microenvironment by regulating CD8+ T cell cytotoxicity and chemokine expression, specifically downregulating CXCL9 and CXCL10, which are critical for T cell recruitment. Our findings reveal a novel regulatory axis whereby SNHG26 promotes CRC progression by destabilising CDKN2A mRNA, resulting in enhanced cell proliferation, cuproptosis and immune evasion. This study provides new insights into the molecular mechanisms underlying CRC development.

**Abbreviations**
CCK‐8: cell counting kit‐8
CDKN2A: cyclin‐dependent kinase inhibitor 2A
CRC: colorectal cancer
Cu + ELES: copper plus elesclomol (cuproptosis inducer)
CXCL9/10: C‐X‐C motif chemokine ligand 9/10
ELES: elesclomol
IL‐2: interleukin‐2
lncRNA: long non‐coding RNA
PBMCs: peripheral blood mononuclear cells
RIP: RNA immunoprecipitation
RIPA: radioimmunoprecipitation assay
shRNA: short hairpin RNA
siRNA: small interfering RNA
SNHG26: small nucleolar RNA host gene 26

## 1 Introduction

Colorectal cancer (CRC) remains one of the most prevalent malignancies worldwide, ranking as the third most commonly diagnosed cancer and the second leading cause of cancer‐related mortality globally [1]. Despite significant advances in diagnostic techniques and therapeutic strategies, the prognosis for patients with advanced CRC remains poor, largely due to tumour metastasis and therapy resistance [2]. Understanding the molecular mechanisms underlying CRC progression is therefore important for developing more effective treatment approaches.

Long non‐coding RNAs (lncRNAs), defined as RNA transcripts longer than 200 nucleotides with limited protein‐coding potential, have emerged as critical regulators in various biological processes, including cancer development and progression [3, 4]. Accumulating evidence indicates that lncRNAs can function as oncogenes or tumour suppressors by modulating gene expression at epigenetic, transcriptional and post‐transcriptional levels [5, 6]. In CRC, several lncRNAs have been identified as key players in regulating cell proliferation, apoptosis, metastasis and drug resistance [7, 8]. LncRNAs exert their biological functions through diverse mechanisms. They may act as scaffolds or guides to recruit proteins to specific genomic loci, as decoys to sequester regulatory proteins away from their targets, or as competitive endogenous RNAs to sponge microRNAs [9]. Moreover, lncRNAs can directly interact with mRNAs to regulate their stability and translation efficiency [10]. These versatile roles position lncRNAs as integral components of the complex regulatory networks governing cellular processes and disease states.

Small nucleolar RNA host gene 26 (SNHG26), a recently identified lncRNA, has been reported to be aberrantly expressed in various human cancers [11, 12]. However, its role and regulatory mechanisms in CRC remain largely unexplored. Preliminary studies have suggested that SNHG26 may participate in cancer cell proliferation and metastasis, but the precise molecular pathways through which SNHG26 exerts its functions in CRC are not well defined.

The tumour microenvironment plays a important role in cancer progression and therapeutic response [13, 14]. Cytotoxic CD8+ T cells represent a major component of the anti‐tumour immune response, with their infiltration into tumour tissues often correlating with improved patient outcomes [15, 16]. However, tumours can employ various mechanisms to evade immune surveillance, including the suppression of T cell recruitment and function. Recent studies have implicated lncRNAs in modulating tumour‐immune interactions [17], but the potential role of SNHG26 in regulating the immune microenvironment in CRC has not been investigated.

In this study, we investigated the expression pattern and biological functions of SNHG26 in CRC. We found that SNHG26 was significantly upregulated in CRC tissues and cell lines, and its high expression was associated with poor prognosis. Functional experiments revealed that SNHG26 promoted CRC cell proliferation and conferred resistance to cell death. Mechanistically, we demonstrated that SNHG26 established a previously unrecognised regulatory axis with CDKN2A in CRC. Furthermore, we explored the immunomodulatory effects of this axis on CD8+ T cell‐mediated anti‐tumour immunity. Our findings provide novel insights into the molecular mechanisms underlying CRC progression and suggest that targeting SNHG26 could be a promising therapeutic strategy for CRC treatment.

## 2 Materials and Methods

### 2.1 Cell Lines and Culture Conditions

Colorectal cancer cell lines (HCT116, SW480, SW620, HT29 and DLD1) and the normal colon epithelial cell line Fetal Human Colon (FHC) were obtained from the American Type Culture Collection (ATCC, Manassas, VA, USA). All cancer cell lines were maintained in Dulbecco's Modified Eagle's Medium (DMEM, Gibco, Thermo Fisher Scientific, Waltham, MA, USA) supplemented with 10% fetal bovine serum (FBS, Biological Industries, Cromwell, CT, USA) and 1% penicillin/streptomycin (Gibco). FHC cells were cultured in DMEM medium supplemented with 10% FBS, 10 ng/mL cholera toxin, 5 μg/mL insulin, 5 μg/mL transferrin and 100 ng/mL hydrocortisone. All cells were cultured at 37°C in a humidified incubator with 5% CO_2_.

### 2.2 Patient Samples

A total of 23 pairs of CRC tissues and adjacent normal tissues were collected from patients who underwent surgical resection at the Second Affiliated Hospital of Army Medical University. None of the patients received chemotherapy or radiotherapy before surgery. Tissue samples were immediately snap‐frozen in liquid nitrogen after resection and stored at −80°C until use. Written informed consent was obtained from all patients, and the study was approved by the Ethics Committee of Zhejiang Provincial People's Hospital (Affiliated People's Hospital), Hangzhou Medical College.

### 2.3 RNA Extraction and Quantitative Real‐Time PCR (qRT‐PCR)

Total RNA was extracted from tissues and cells using TRIzol reagent (Invitrogen, Carlsbad, CA, USA) according to the manufacturer's instructions. RNA concentration and purity were determined using a NanoDrop 2000 spectrophotometer (Thermo Fisher Scientific). For qRT‐PCR, 1 μg of total RNA was reverse‐transcribed into cDNA using the PrimeScript RT Reagent Kit (TaKaRa, Shiga, Japan). qRT‐PCR was performed on a CFX96 Real‐Time PCR Detection System (Bio‐Rad, Hercules, CA, USA) using SYBR Premix Ex Taq II (TaKaRa). The cycling conditions were as follows: initial denaturation at 95°C for 30 s, followed by 40 cycles of 95°C for 5 s and 60°C for 30 s. Relative expression levels were calculated using the 2^−ΔΔCt^ method with GAPDH as an internal control.

### 2.4 Small Interfering RNA (siRNA), shRNA and Plasmid Transfection

siRNAs targeting SNHG26 (si‐SNHG26#1 and si‐SNHG26#2) and negative control siRNA (si‐NC) were designed and synthesised by RiboBio (Guangzhou, China). The SNHG26 overexpression plasmid was constructed by GenePharma (Shanghai, China). Cell transfection was performed using Lipofectamine 3000 (Invitrogen) following the manufacturer's protocol. For siRNA transfection, cells were seeded in 6‐well plates and transfected with 50 nM siRNA when they reached 50%–60% confluence. For plasmid transfection, 2 μg of plasmid DNA was used per well in 6‐well plates. Transfection efficiency was evaluated by qRT‐PCR or western blotting 48 h post‐transfection.

For stable CDKN2A knockdown, shRNA targeting CDKN2A (sh‐CDKN2A) and negative control shRNA (sh‐NC) were designed and constructed into the lentiviral vector pLKO.1 (Addgene, Watertown, MA, USA). Lentiviral particles were produced in HEK293T cells by co‐transfecting the shRNA‐expressing plasmid, psPAX2 packaging plasmid and pMD2.G envelope plasmid using Lipofectamine 3000. Viral supernatants were collected 48 and 72 h post‐transfection, filtered through a 0.45 μm filter and used to infect target cells with 8 μg/mL polybrene (Sigma‐Aldrich, St. Louis, MO, USA). After 48 h of infection, stable cell lines were selected using 2 μg/mL puromycin (Invitrogen) for 7 days. For double‐stable cell lines, SNHG26‐overexpressing cells were further infected with sh‐CDKN2A or sh‐NC lentivirus and selected with both puromycin and G418 (500 μg/mL, Gibco). Knockdown efficiency was confirmed by western blotting and qRT‐PCR.

### 2.5 Cell Viability Assay

Cell viability was assessed using the Cell Counting Kit‐8 (CCK‐8, Dojindo, Kumamoto, Japan). Briefly, cells were seeded in 96‐well plates at a density of 3 × 10^3^ cells per well. After attachment, cells were treated as indicated in the figure legends. At specified time points, 10 μL of CCK‐8 solution was added to each well, followed by incubation at 37°C for 2 h. Absorbance was measured at 450 nm using a microplate reader (Bio‐Rad). For Cu + ELES (copper plus elesclomol) treatments, cells were exposed to various concentrations (0–1000 nM) of Cu + ELES for the indicated durations.

### 2.6 Colony Formation Assay

Cells were seeded in 6‐well plates at a density of 1 × 10^3^ cells per well and cultured for 14 days. The medium was changed every 3 days. Colonies were fixed with 4% paraformaldehyde for 15 min and stained with 0.1% crystal violet for 20 min. After washing with PBS, colonies containing more than 50 cells were counted under a microscope. Images were captured using a digital camera, and colonies were quantified using ImageJ software (National Institutes of Health, Bethesda, MD, USA).

### 2.7 EdU Incorporation Assay

Cell proliferation was evaluated using the EdU (5‐ethynyl‐2′‐deoxyuridine) Cell Proliferation Kit (RiboBio) according to the manufacturer's instructions. Briefly, cells were seeded in 24‐well plates with coverslips and incubated with 50 μM EdU for 2 h at 37°C. After fixation with 4% paraformaldehyde and permeabilisation with 0.5% Triton X‐100, cells were incubated with Apollo reaction cocktail for 30 min. Cell nuclei were counterstained with Hoechst 33342. Images were acquired using a fluorescence microscope (Olympus, Tokyo, Japan), and the percentage of EdU‐positive cells was calculated from five random fields.

### 2.8 Flow Cytometry Analysis of Apoptosis

Apoptosis was detected using the Annexin V‐FITC/PI Apoptosis Detection Kit (BD Biosciences, San Jose, CA, USA) according to the manufacturer's instructions. Briefly, cells were harvested, washed twice with cold PBS and resuspended in 1× binding buffer. Then, 5 μL of Annexin V‐FITC and 5 μL of propidium iodide (PI) were added to 100 μL of cell suspension, followed by incubation in the dark for 15 min at room temperature. After adding 400 μL of binding buffer, the samples were analysed by flow cytometry (BD FACSCanto II). A minimum of 10,000 events were collected for each sample. The data were analysed using FlowJo software (Tree Star, Ashland, OR, USA).

### 2.9 Western Blotting

Cells were lysed in radioimmunoprecipitation assay (RIPA) buffer (Beyotime, Shanghai, China) containing protease inhibitor cocktail (Roche, Basel, Switzerland). Protein concentration was determined using the BCA Protein Assay Kit (Thermo Fisher Scientific). Equal amounts of protein (30 μg) were separated by 10% SDS‐PAGE and transferred to PVDF membranes (Millipore, Burlington, MA, USA). After blocking with 5% non‐fat milk in TBST for 1 h at room temperature, the membranes were incubated with primary antibodies overnight at 4°C, followed by incubation with HRP‐conjugated secondary antibodies for 1 h at room temperature. The following primary antibodies were used: anti‐CDKN2A (1:1000, Cell Signaling Technology, #4824, Danvers, MA, USA), anti‐GAPDH (1:5000, Proteintech, #60004‐1‐Ig, Rosemont, IL, USA). Protein bands were visualised using enhanced chemiluminescence reagents (Millipore) and imaged using the ChemiDoc XRS+ system (Bio‐Rad). Band intensities were quantified using ImageJ software and normalised to GAPDH.

### 2.10 Subcellular Fractionation

Cytoplasmic and nuclear fractions were isolated using the PARIS Kit (Thermo Fisher Scientific) according to the manufacturer's instructions. Briefly, cells were harvested, washed with PBS and resuspended in cell fractionation buffer. After incubation on ice for 10 min, the suspension was centrifuged at 500× *g* for 5 min at 4°C. The supernatant (cytoplasmic fraction) was collected and the nuclear pellet was washed and lysed in cell disruption buffer. RNA was extracted from both fractions for qRT‐PCR analysis. GAPDH and U6 were used as cytoplasmic and nuclear markers, respectively.

### 2.11 RNA Immunoprecipitation (RIP)

RIP assays were performed using the Magna RIP RNA‐Binding Protein Immunoprecipitation Kit (Millipore) according to the manufacturer's instructions. Briefly, cells were lysed in RIP lysis buffer containing protease and RNase inhibitors. Cell lysates were incubated with magnetic beads conjugated with anti‐CDKN2A antibody or normal rabbit IgG (negative control) at 4°C overnight. After washing, the immunoprecipitated RNA was extracted, purified and analysed by qRT‐PCR. Input samples (10% of cell lysates) were used as positive controls. The results were expressed as fold enrichment relative to the IgG control.

### 2.12 RNA Pull‐Down Assay

Biotin‐labelled SNHG26 and antisense SNHG26 were synthesised by RiboBio. Cell lysates were prepared using RIPA buffer and incubated with biotinylated probes at 4°C overnight. Streptavidin‐coupled magnetic beads (Invitrogen) were added and incubated for an additional 4 h. After washing with RIPA buffer, the bound proteins were eluted and analysed by RNA sequencing or western blotting. For RNA sequencing, libraries were prepared using the NEBNext Ultra RNA Library Prep Kit for Illumina (New England Biolabs, Ipswich, MA, USA) and sequenced on an Illumina NovaSeq 6000 platform.

### 2.13 RNA Stability Assay

Cells were treated with actinomycin D (5 μg/mL, Sigma‐Aldrich) to inhibit transcription. Total RNA was extracted at 0‐, 2‐, 4‐ and 6‐h post‐treatment. The remaining CDKN2A mRNA was quantified by qRT‐PCR and normalised to 18S rRNA. The half‐life of CDKN2A mRNA was calculated as the time required for a 50% reduction in mRNA levels after actinomycin D treatment.

### 2.14 CD8+ T Cell Isolation and Co‐Culture Assay

Peripheral blood mononuclear cells (PBMCs) were isolated from healthy donors (*n* = 5) using Ficoll‐Paque density gradient centrifugation. CD8+ T cells were purified from PBMCs using the CD8+ T Cell Isolation Kit (Miltenyi Biotec, Bergisch Gladbach, Germany) according to the manufacturer's instructions. The purity of isolated CD8+ T cells was verified by flow cytometry (≥ 95%). CD8+ T cells were purified from PBMCs using the CD8+ T Cell Isolation Kit (Miltenyi Biotec, Bergisch Gladbach, Germany) according to the manufacturer's instructions. The purity of isolated CD8+ T cells was verified by flow cytometry (≥ 95%). CD8+ T cells were activated with anti‐CD3/CD28 beads (Gibco) and 100 U/mL IL‐2 for 48 h before co‐culture experiments. For co‐culture assays, activated CD8+ T cells were co‐cultured with CRC cells at different effector‐to‐target ratios (2:1, 3:1 and 5:1) for 24 h. Cytotoxicity was measured using the CytoTox 96 Non‐Radioactive Cytotoxicity Assay (Promega, Madison, WI, USA) according to the manufacturer's instructions. The cytotoxicity ratio was calculated as the percentage of specific lysis relative to maximum lysis.

### 2.15 Migration Assay

Cell migration was assessed using Transwell chambers (8 μm pore size, Corning, Corning, NY, USA). Briefly, 2 × 10^4^ cells in serum‐free medium were seeded in the upper chamber, while the lower chamber was filled with medium containing 20% FBS as a chemoattractant. After 24 h of incubation, cells that had migrated to the lower surface of the membrane were fixed with 4% paraformaldehyde and stained with 0.1% crystal violet. Images were captured from five random fields under a microscope, and the number of migrated cells was counted. The migration index was calculated as the ratio of the number of migrated cells in the experimental group to that in the control group.

### 2.16 Statistical Analysis

All experiments were performed at least three times independently, and data are presented as mean ± standard deviation (SD). Statistical analyses were performed using GraphPad Prism 9.0 software (GraphPad Software, San Diego, CA, USA). Differences between two groups were analysed using Student's *t*‐test. Comparisons among multiple groups were performed using one‐way ANOVA followed by Tukey's post hoc test. Correlation analyses were conducted using Pearson's correlation coefficient. Survival analysis was conducted using the Kaplan–Meier method and log‐rank test. All experiments were performed with a minimum of three biological replicates (*n* ≥ 3) unless otherwise specified in the figure legends. *p* < 0.05 was considered statistically significant.

## 3 Results

### 3.1 SNHG26 Is Upregulated in Colorectal Cancer and Its Knockdown Inhibits Proliferation

**FIGURE 1:**
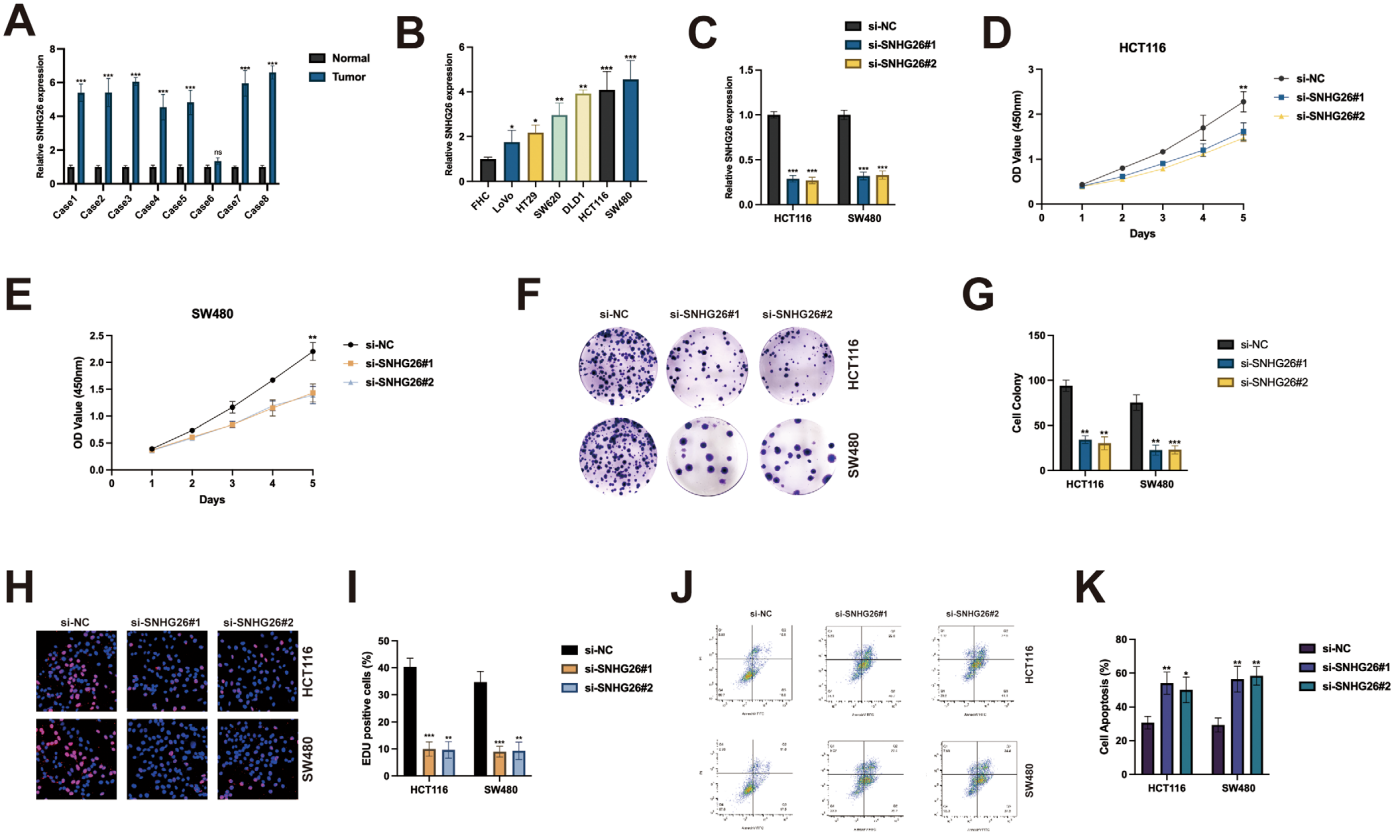
SNHG26 is upregulated in colorectal cancer and knockdown inhibits proliferation. (A) Relative SNHG26 expression in colorectal cancer tissues compared to adjacent normal tissues across eight cohorts. (B) Relative SNHG26 expression in colorectal cancer cell lines (HT29, SW620, DLD1, HCT116 and SW480) and normal colon epithelial cell line FHC. (C) Knockdown efficiency of SNHG26 using two independent siRNAs (si‐SNHG26#1 and si‐SNHG26#2) in HCT116 and SW480 cells. (D, E) Cell proliferation assessed by CCK‐8 assay in HCT116 cells (D) and SW480 cells (E) after SNHG26 knockdown. (F, G) Colony formation assay results in HCT116 and SW480 cells following SNHG26 knockdown. Representative images (F) and quantification (G) are shown. (H, I) EdU incorporation assay showing DNA synthesis in HCT116 and SW480 cells after SNHG26 knockdown. Representative images (H) and quantification (I) are shown. (J‐K) Flow cytometry analysis of apoptosis in HCT116 and SW480 cells after SNHG26 knockdown. Representative plots (J) and quantification (K) are shown. *p < 0.05, **p < 0.01, ***p < 0.001.

To elucidate the clinical significance of SNHG26 in CRC pathogenesis, we first performed comprehensive expression analyses across multiple independent cohorts. SNHG26 expression was significantly elevated in CRC tissues compared to adjacent normal tissues (Figure 1A, *p* < 0.001). This upregulation was consistently observed across all eight examined cohorts, suggesting a robust association between SNHG26 expression and CRC development. Extending these observations to in vitro models, we assessed SNHG26 expression in a panel of CRC cell lines (HT29, SW620, DLD1, HCT116 and SW480) relative to the normal colon epithelial cell line FHC. All cancer cell lines exhibited significantly higher SNHG26 expression compared to FHC cells, with HCT116 and SW480 displaying the most pronounced elevation (Figure 1B). Based on these expression profiles, HCT116 and SW480 cells were selected for subsequent functional investigations. To elucidate the biological significance of SNHG26 in CRC, we employed RNA interference technology using two independent siRNAs targeting distinct regions of SNHG26. Quantitative RT‐PCR analysis confirmed that both siRNAs effectively suppressed SNHG26 expression by more than 70% in both HCT116 and SW480 cells (Figure 1C), validating the specificity and efficiency of the knockdown approach. Functional characterisation revealed that SNHG26 silencing significantly attenuated cell proliferation in both cell lines, as evidenced by CCK‐8 growth curve assays (Figure 1D,E). This anti‐proliferative effect was further corroborated by colony formation assays, which demonstrated a substantial reduction (approximately 60%) in the clonogenic potential of SNHG26‐depleted cells (Figure 1F,G). To directly assess DNA synthesis, we performed EdU incorporation assays, which showed a marked decrease in the proportion of proliferating cells following SNHG26 knockdown—from approximately 45% to 20% in HCT116 cells and from 50% to 25% in SW480 cells (Figure 1H,I).

In parallel with reduced proliferation, flow cytometric analysis revealed that SNHG26 depletion significantly enhanced apoptosis, increasing the apoptotic fraction from approximately 5% to 25% in HCT116 cells and from 7% to 30% in SW480 cells (Figure 1J,K). Collectively, these data establish that SNHG26 plays a critical role in sustaining CRC cell proliferation and survival, with its depletion resulting in profound anti‐tumorigenic effects.

### 3.2 SNHG26 Knockdown Enhances Sensitivity to Cuproptosis

**FIGURE 2:**
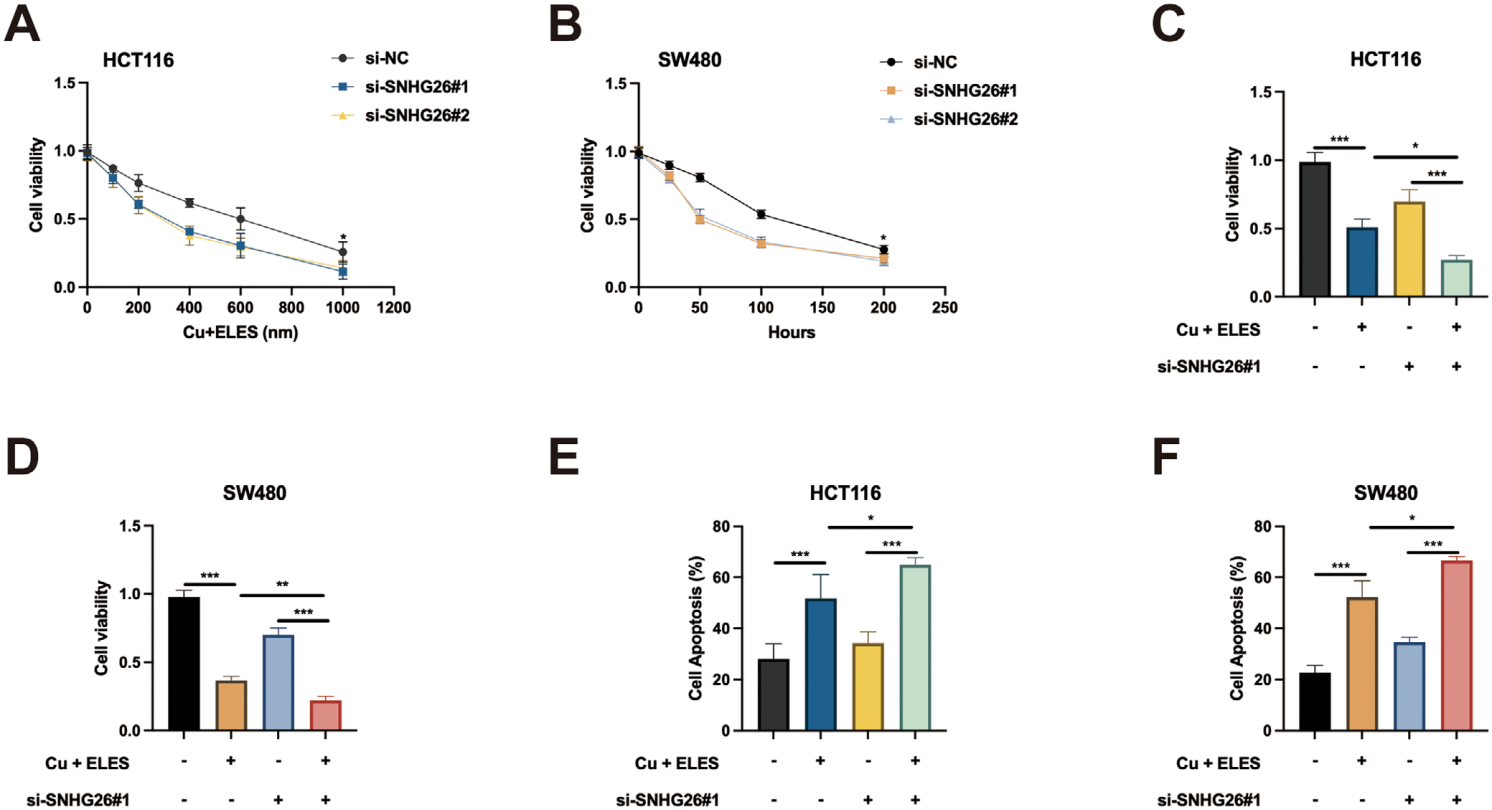
SNHG26 knockdown enhances sensitivity to Cu + ELES‐induced cuproptosis. (A, B) Cell viability assays showing the effect of SNHG26 knockdown on sensitivity to Cu + ELES treatment in HCT116 cells (A) and SW480 cells (B) in a dose‐dependent and time‐dependent manner, respectively. (C, D) Combination treatment showing the synergistic effect of SNHG26 knockdown and Cu + ELES treatment on cell viability in HCT116 cells (C) and SW480 cells (D). (E, F) Flow cytometry analysis of apoptosis following Cu + ELES treatment with or without SNHG26 knockdown in HCT116 cells (E) and SW480 cells (F). *p < 0.05, **p < 0.01, ***p < 0.001.

Given the emerging role of cuproptosis in cancer therapeutic strategies and the relationship between SNHG26 and cuproptosis in CRC [18], we investigated whether SNHG26 modulates the sensitivity of CRC cells to this novel cell death pathway. We employed Cu + ELES, a well‐characterised cuproptosis inducer, and evaluated its efficacy in SNHG26‐depleted cells. Dose–response analyses revealed that SNHG26 knockdown significantly enhanced the sensitivity of both HCT116 and SW480 cells to Cu + ELES treatment (Figure 2A). This sensitisation effect exhibited a clear dose‐dependency, becoming particularly pronounced at concentrations exceeding 600 nM. In HCT116 cells, while control cells maintained approximately 40% viability at 1000 nM Cu + ELES, SNHG26‐depleted cells displayed less than 20% viability at the same concentration. Similarly, time‐course experiments demonstrated that SNHG26 knockdown accelerated the kinetics of Cu + ELES‐induced cytotoxicity, with significantly reduced viability observed at earlier time points compared to control cells (Figure 2B). To assess potential synergistic interactions, we conducted combination treatment studies. Remarkably, while SNHG26 knockdown or Cu + ELES treatment alone induced moderate cytotoxicity (approximately 30% and 40% reduction in viability, respectively), their combination resulted in a profound synergistic effect, reducing cell viability by more than 70% in both cell lines (Figure 2C,D). This synergism was further validated by apoptosis assays, which showed that the combination of SNHG26 knockdown and Cu + ELES treatment significantly augmented apoptotic rates compared to either intervention alone—from approximately 50% with Cu + ELES alone to over 65% in HCT116 cells and from 55% to nearly 70% in SW480 cells (Figure 2E,F). These findings demonstrate that SNHG26 confers protection against cuproptosis in CRC cells, and its depletion substantially enhances the therapeutic efficacy of Cu + ELES, suggesting a previously unrecognised role for SNHG26 in modulating copper‐dependent cell death pathways.

### 3.3 SNHG26 Directly Interacts With CDKN2A

**FIGURE 3:**
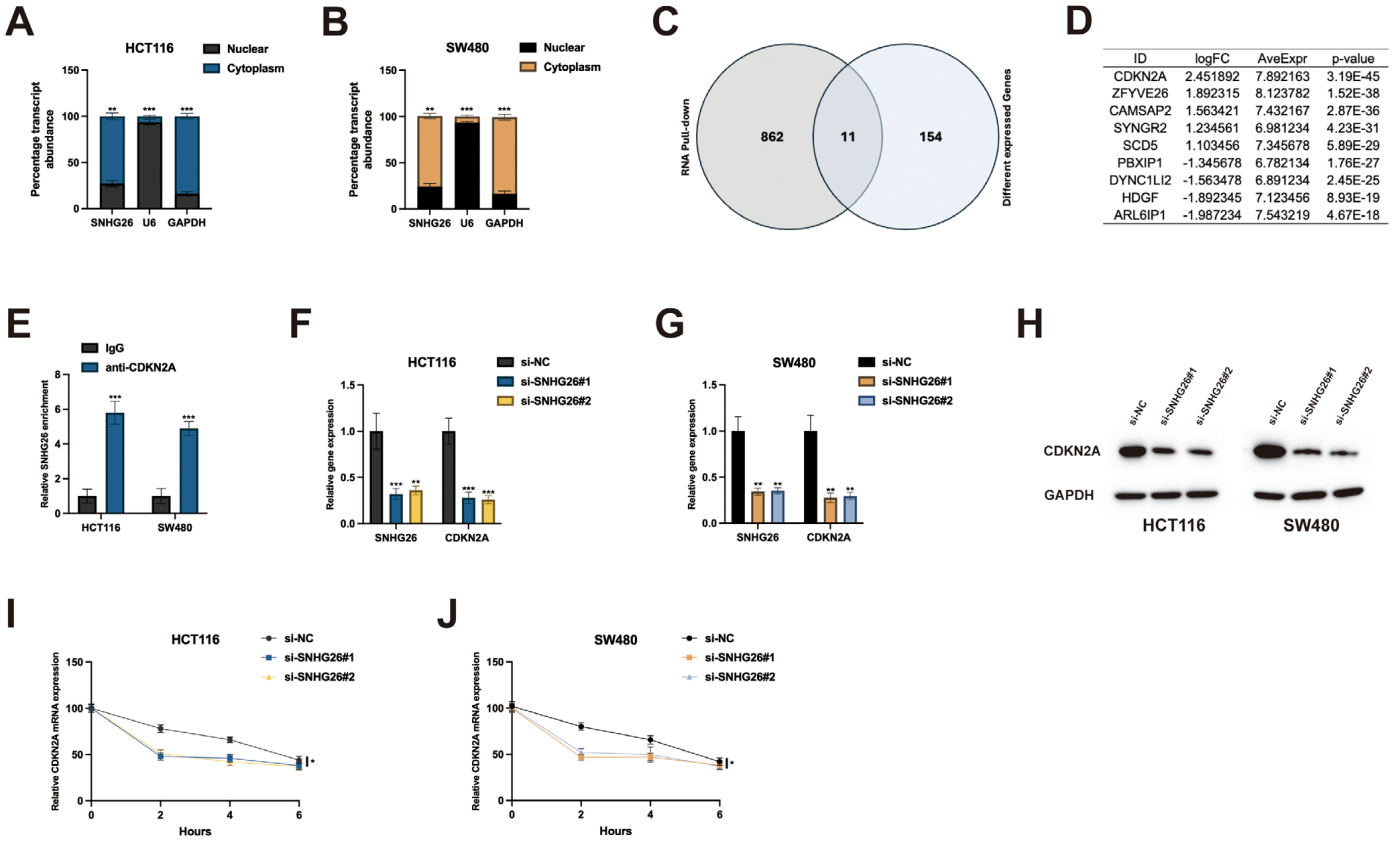
SNHG26 interacts with CDKN2A. (A, B) Subcellular localisation of SNHG26 determined by qRT‐PCR analysis after nuclear and cytoplasmic fractionation in HCT116 cells (A) and SW480 cells (B). U6 and GAPDH served as nuclear and cytoplasmic markers, respectively. (C) Venn diagram showing the overlap between RNA pull‐down and differentially expressed gene datasets. (D) Table showing the top differentially expressed genes identified by RNA‐seq with their corresponding log fold change, average expression and *p* values. (E) RNA immunoprecipitation (RIP) assay confirming the interaction between SNHG26 and CDKN2A in HCT116 and SW480 cells. (F, G) qRT‐PCR analysis of SNHG26 and CDKN2A expression in HCT116 cells (F) and SW480 cells (G) after SNHG26 knockdown. (H) Western blot analysis of CDKN2A protein expression after SNHG26 knockdown in HCT116 and SW480 cells. (I, J) RNA stability assay showing the effect of SNHG26 knockdown on CDKN2A mRNA decay in HCT116 cells (I) and SW480 cells (J). *p < 0.05, **p < 0.01, ***p < 0.001.

To delineate the molecular mechanisms underlying SNHG26 function, we first determined its subcellular distribution. Subcellular fractionation followed by qRT‐PCR analysis revealed predominant cytoplasmic localisation of SNHG26 in both HCT116 and SW480 cells (> 70% of total SNHG26, Figure 3A,B), with U6 and GAPDH serving as reliable nuclear and cytoplasmic markers, respectively. This cytoplasmic enrichment suggested that SNHG26 might function at the post‐transcriptional level through interactions with cytoplasmic components. To identify potential SNHG26‐interacting partners, we employed an unbiased approach combining RNA pull‐down with high‐throughput RNA sequencing. Comparative analysis of pull‐down and knockdown datasets identified 11 genes that were both physically associated with SNHG26 and differentially expressed upon SNHG26 depletion (Figure 3C). Among these candidates, CDKN2A exhibited the most significant association, as evidenced by the highest fold enrichment and lowest *p* value (Figure 3D).

To validate this interaction, we performed RNA immunoprecipitation (RIP) assays using an anti‐CDKN2A antibody. Quantitative RT‐PCR analysis of immunoprecipitated RNA confirmed significant enrichment of SNHG26 in CDKN2A immunoprecipitates compared to IgG control in both HCT116 and SW480 cells (Figure 3E), substantiating the direct interaction between SNHG26 and CDKN2A. Functional relevance of this interaction was established by examining CDKN2A expression following SNHG26 modulation. SNHG26 knockdown significantly upregulated CDKN2A mRNA levels by approximately 2.5‐fold in both cell lines (Figure 3F,G), with corresponding increases in protein expression (Figure 3H). These findings suggested that SNHG26 negatively regulates CDKN2A expression. To elucidate the underlying mechanism, we conducted RNA stability assays using actinomycin D to inhibit de novo transcription. Notably, SNHG26 depletion significantly prolonged CDKN2A mRNA half‐life from approximately 3 h in control cells to 5 h in SNHG26‐knockdown cells (Figure 3I,J). This stabilisation effect was consistent across both cell lines, indicating that SNHG26 promotes CDKN2A mRNA degradation, thereby suppressing its expression.

### 3.4 CDKN2A Exhibits Context‐Dependent Pro‐Tumorigenic Effects in CRC

**FIGURE 4:**
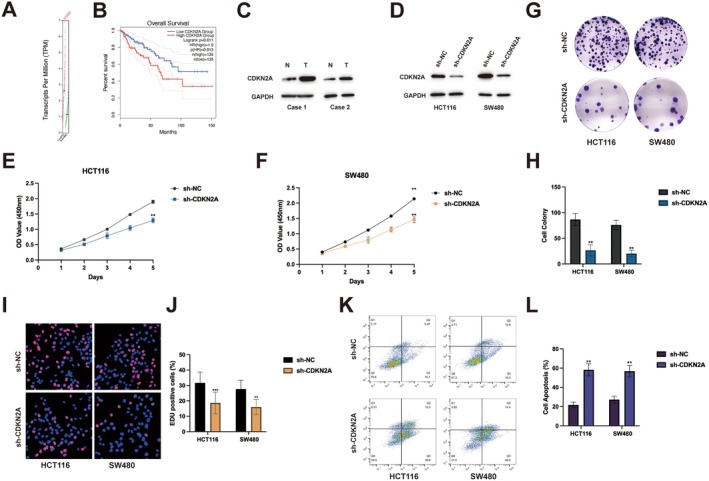
CDKN2A functions as an oncogene in colorectal cancer. (A) TCGA data analysis of CDKN2A expression in colorectal cancer tissues versus normal tissues. (B) Kaplan–Meier survival analysis of colorectal cancer patients stratified by CDKN2A expression levels. (C) Western blot analysis of CDKN2A protein levels in paired tumour (T) and adjacent normal (N) tissues from clinical samples. (D) Western blot confirming CDKN2A knockdown efficiency in HCT116 and SW480 cells. (E, F) Cell proliferation assessed by CCK‐8 assay in HCT116 cells (E) and SW480 cells (F) after CDKN2A knockdown. (G, H) Colony formation assay results in HCT116 and SW480 cells following CDKN2A knockdown. Representative images (G) and quantification (H) are shown. (I, J) EdU incorporation assay showing DNA synthesis in HCT116 and SW480 cells after CDKN2A knockdown. Representative images (I) and quantification (J) are shown. (K, L) Flow cytometry analysis of apoptosis in HCT116 and SW480 cells after CDKN2A knockdown. Representative plots (K) and quantification (L) are shown. **p < 0.01, ***p < 0.001.

Having identified CDKN2A as a direct target of SNHG26, we next investigated its context‐dependent functional role in CRC. Analysis of The Cancer Genome Atlas (TCGA) data revealed significant upregulation of CDKN2A in CRC tissues compared to normal tissues (Figure 4A, *p* < 0.001). Analysis of TCGA data suggested a trend toward inferior overall survival in patients with elevated CDKN2A expression (Figure 4B, *p* < 0.01, HR = 1.85, 95% CI: 1.25–2.74), indicating potential prognostic significance that warrants validation in larger cohorts. To validate these clinical observations, we examined CDKN2A protein expression in our cohort of paired tumour and adjacent normal tissues. Consistent with the TCGA data, western blot analysis demonstrated markedly higher CDKN2A protein levels in tumour tissues compared to their normal counterparts (Figure 4C), confirming the clinical relevance of CDKN2A overexpression in CRC. To elucidate the functional role of CDKN2A, we established stable CDKN2A‐knockdown cell lines using lentivirus‐delivered shRNA. Western blot analysis confirmed efficient silencing of CDKN2A protein expression in both HCT116 and SW480 cells (Figure 4D). Surprisingly, despite its established role as a tumour suppressor in many contexts, CDKN2A depletion significantly inhibited CRC cell proliferation, as evidenced by CCK‐8 assays (Figure 4E,F). This anti‐proliferative effect was further substantiated by colony formation assays, which demonstrated a substantial reduction (approximately 65%) in clonogenic potential following CDKN2A knockdown (Figure 4G,H). EdU incorporation assays provided direct evidence that CDKN2A silencing impaired DNA synthesis, reducing the proportion of EdU‐positive cells from approximately 45% to 18% in HCT116 cells and from 50% to 20% in SW480 cells (Figure 4I,J). Concomitantly, flow cytometric analysis revealed that CDKN2A knockdown significantly enhanced apoptosis, increasing the apoptotic fraction from approximately 5% to 30% in HCT116 cells and from 7% to 35% in SW480 cells (Figure 4K,L).

### 3.5 CDKN2A Mediates the Oncogenic Effects of SNHG26 in Colorectal Cancer

**FIGURE 5:**
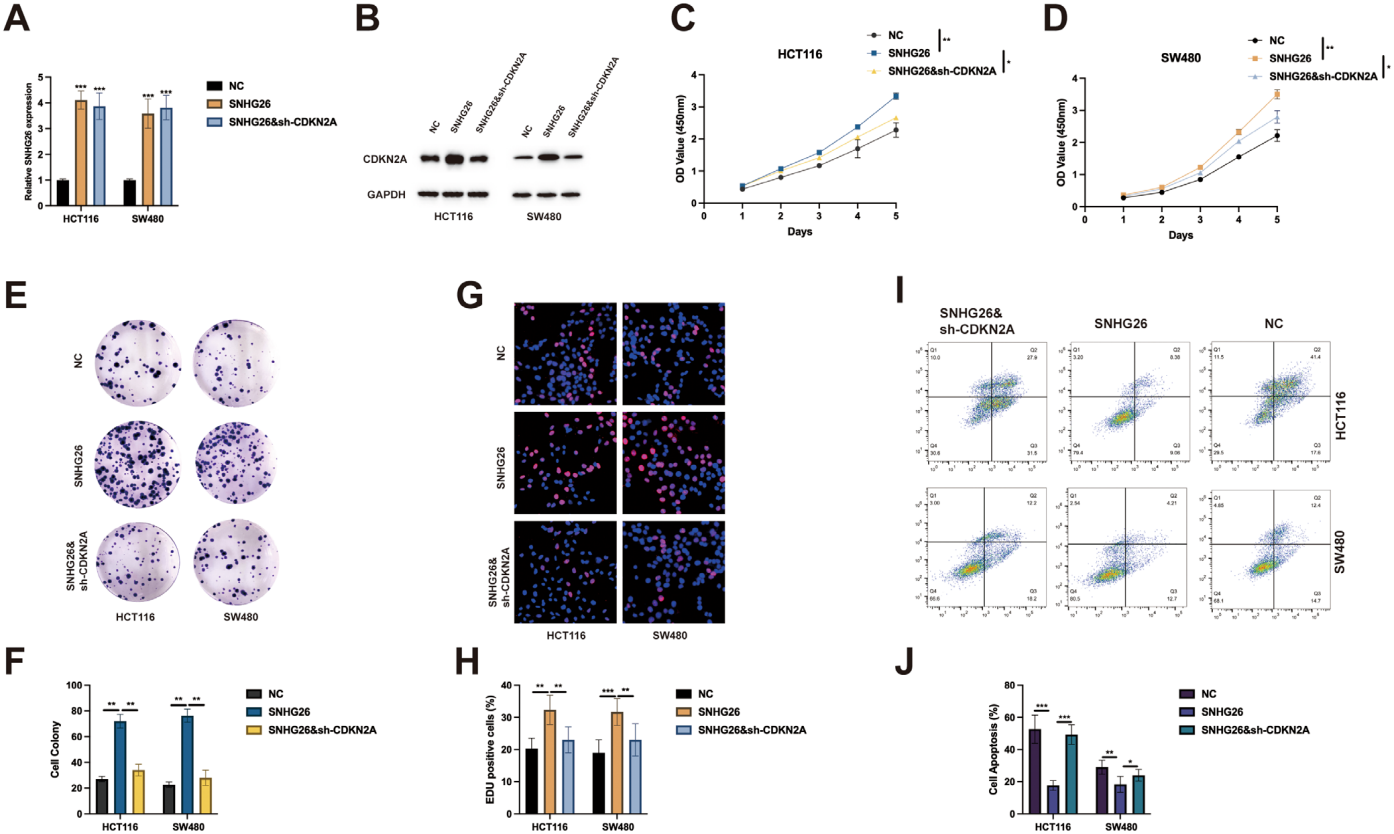
CDKN2A knockdown rescues the effects of SNHG26. (A) qRT‐PCR analysis confirming SNHG26 overexpression in HCT116 and SW480 cells. (B) Western blot analysis confirming CDKN2A knockdown in SNHG26‐overexpressing HCT116 and SW480 cells. (C, D) Cell proliferation assessed by CCK‐8 assay in HCT116 cells (C) and SW480 cells (D) with SNHG26 overexpression and/or CDKN2A knockdown. (E, F) Colony formation assay results in HCT116 and SW480 cells with SNHG26 overexpression and/or CDKN2A knockdown. Representative images (E) and quantification (F) are shown. (G, H) EdU incorporation assay in HCT116 and SW480 cells with SNHG26 overexpression and/or CDKN2A knockdown. Representative images (G) and quantification (H) are shown. (I, J) Flow cytometry analysis of apoptosis in HCT116 and SW480 cells with SNHG26 overexpression and/or CDKN2A knockdown. Representative plots (I) and quantification (J) are shown. *p < 0.05, **p < 0.01, ***p < 0.001.

To establish the functional interrelationship between SNHG26 and CDKN2A, we generated stable cell lines with SNHG26 overexpression alone or in combination with CDKN2A knockdown. Quantitative RT‐PCR confirmed successful SNHG26 overexpression, with approximately fourfold increase in expression compared to control cells (Figure 5A). Immunoblot analysis verified effective CDKN2A depletion in the double‐manipulated cells (Figure 5B). Comprehensive functional characterisation revealed that SNHG26 overexpression significantly enhanced cell proliferation in both HCT116 and SW480 cells, while concurrent CDKN2A knockdown substantially attenuated this pro‐proliferative effect (Figure 5C,D). Similarly, clonogenic assays demonstrated that SNHG26 overexpression increased colony formation by approximately 70% in both cell lines, an effect that was reduced to approximately 30% following concomitant CDKN2A depletion (Figure 5E,F). EdU incorporation assays further corroborated these findings, showing that SNHG26 overexpression enhanced DNA synthesis, increasing the percentage of EdU‐positive cells from approximately 45% to 70% in HCT116 cells and from 50% to 75% in SW480 cells. This proliferative advantage was significantly diminished by concurrent CDKN2A knockdown, which reduced EdU incorporation to approximately 55% in both cell lines (Figure 5G,H). Consistent with these observations, flow cytometric analysis revealed that SNHG26 overexpression exerted a potent anti‐apoptotic effect, reducing basal apoptosis rates from approximately 5% to 2% in HCT116 cells and from 7% to 3% in SW480 cells. This cytoprotective effect was partially reversed by CDKN2A silencing, which increased apoptosis rates to approximately 4% in HCT116 cells and 5% in SW480 cells (Figure 5I,J).

### 3.6 SNHG26 Confers Resistance to Cuproptosis Through CDKN2A Upregulation

**FIGURE 6:**
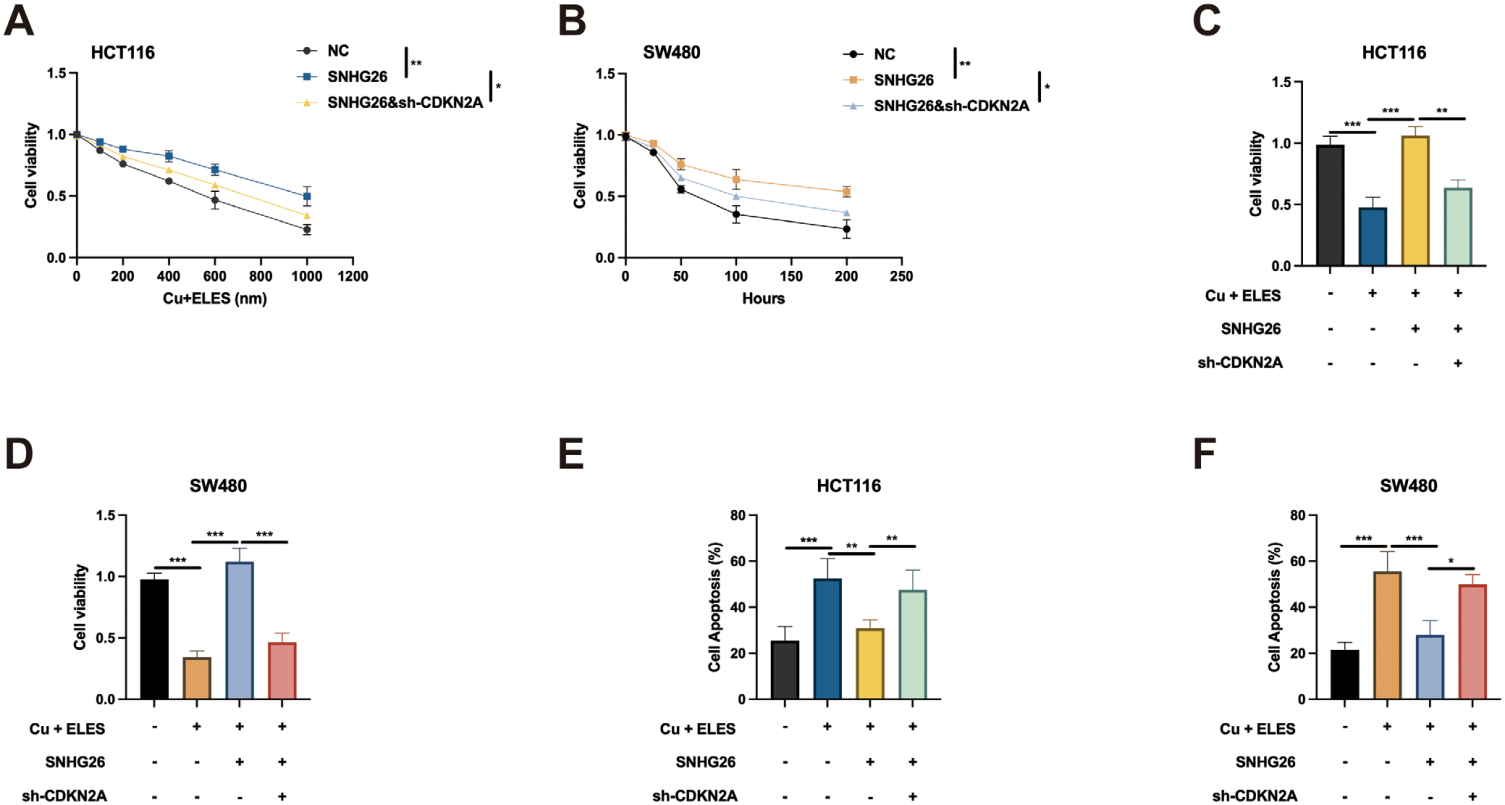
SNHG26 promotes resistance to cuproptosis via CDKN2A regulation. (A, B) Cell viability assays showing the effect of SNHG26 overexpression and/or CDKN2A knockdown on sensitivity to Cu + ELES treatment in HCT116 cells (A) and SW480 cells (B) in a dose‐dependent and time‐dependent manner, respectively. (C, D) Combination treatment showing the effect of SNHG26 overexpression and/or CDKN2A knockdown on cell viability in the presence of Cu + ELES in HCT116 cells (C) and SW480 cells (D). (E, F) Flow cytometry analysis of apoptosis following Cu + ELES treatment in cells with SNHG26 overexpression and/or CDKN2A knockdown in HCT116 cells (E) and SW480 cells (F). *p < 0.05, **p < 0.01, ***p < 0.001.

Having established the functional significance of the SNHG26‐CDKN2A axis in CRC cell survival and proliferation, we next investigated its role in modulating sensitivity to cuproptosis. Dose–response analyses revealed that SNHG26 overexpression conferred significant resistance to Cu + ELES‐induced cytotoxicity in both HCT116 and SW480 cells (Figure 6A). This protective effect exhibited clear dose‐dependency, becoming particularly evident at concentrations exceeding 600 nM Cu + ELES. At the highest Cu + ELES concentration tested (1000 nM), control cells exhibited approximately 30% viability, whereas SNHG26‐overexpressing cells maintained approximately 60% viability, indicating a substantial cytoprotective effect. Notably, concurrent CDKN2A knockdown partially reversed this protection, reducing viability to approximately 45% at 1000 nM Cu + ELES.

Time‐course experiments further demonstrated that SNHG26 overexpression significantly delayed Cu + ELES‐induced cell death kinetics, with SNHG26‐overexpressing cells maintaining significantly higher viability at all time points examined compared to control cells. This temporal protection was partially abrogated by concomitant CDKN2A depletion (Figure 6B). Combination treatment studies provided additional evidence for the SNHG26‐CDKN2A axis in modulating cuproptosis sensitivity. In both HCT116 and SW480 cells, SNHG26 overexpression significantly attenuated Cu + ELES‐induced cytotoxicity, reducing the inhibitory effect from approximately 40% to 20%. Concurrent CDKN2A knockdown partially restored sensitivity to cuproptosis, increasing the inhibitory effect to approximately 30% (Figure 6C,D). Mechanistically, apoptosis assays revealed that SNHG26 overexpression markedly suppressed Cu + ELES‐induced programmed cell death, reducing apoptosis rates from approximately 50% to 30% in HCT116 cells and from approximately 55% to 25% in SW480 cells. This anti‐apoptotic effect was significantly attenuated by CDKN2A knockdown, which increased apoptosis rates to approximately 45% in HCT116 and 50% in SW480 cells (Figure 6E,F).

### 3.7 SNHG26‐CDKN2A Axis Modulates CD8+ T Cell Cytotoxicity and Chemokine Expression

**FIGURE 7:**
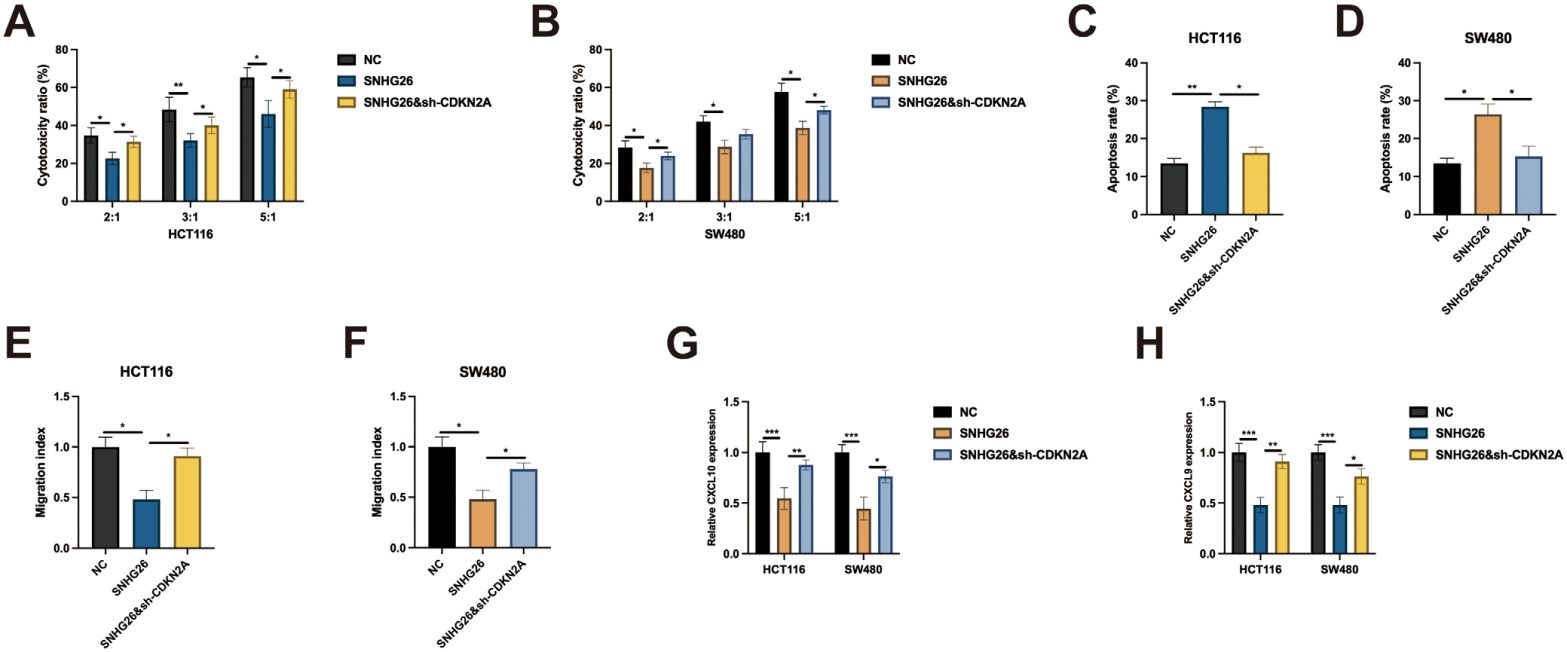
SNHG26‐CDKN2A axis regulates CD8+ T cell cytotoxicity and chemokine expression. (A, B) Cytotoxicity assays showing the effect of SNHG26 overexpression and/or CDKN2A knockdown on CD8+ T cell‐mediated killing of HCT116 cells (A) and SW480 cells (B) at different effector‐to‐target ratios (2:1, 3:1 and 5:1). (C, D) Flow cytometry analysis of apoptosis in CD8+ T cells after co‐culture with HCT116 cells (C) and SW480 cells (D) with SNHG26 overexpression and/or CDKN2A knockdown. (E, F) Migration index of HCT116 cells (E) and SW480 cells (F) with SNHG26 overexpression and/or CDKN2A knockdown. (G, H) qRT‐PCR analysis of CXCL9 (G) and CXCL10 (H) expression in HCT116 and SW480 cells with SNHG26 overexpression and/or CDKN2A knockdown. *p < 0.05, **p < 0.01, ***p < 0.001.

It has been well documented that CDKN2A involves in immune‐related activities in various cancers [19, 20, 21, 22]. To investigate the immunomodulatory functions of the SNHG26‐CDKN2A axis, we established co‐culture systems using activated CD8+ T cells and CRC cells, using activated CD8+ T cells from healthy donors in an allogeneic co‐culture system. Cytotoxicity assays demonstrated that SNHG26 overexpression significantly impaired CD8+ T cell‐mediated killing of both HCT116 and SW480 cells across various effector‐to‐target ratios (Figure 7A,B). At the 5:1 effector‐to‐target ratio, SNHG26 overexpression reduced cytotoxicity from approximately 60% to 35% in HCT116 cells and from 65% to 40% in SW480 cells, indicating substantial immunosuppressive effects. Notably, concurrent CDKN2A knockdown significantly reversed this immunoevasive phenotype, restoring CD8+ T cell cytotoxicity to approximately 55% in HCT116 cells and 60% in SW480 cells at the 5:1 ratio. Similar patterns were observed at lower effector‐to‐target ratios (2:1 and 3:1), confirming the robustness of this immunomodulatory effect across various experimental conditions. Flow cytometric analysis of CD8+ T cells following co‐culture revealed an intriguing reciprocal relationship. SNHG26 overexpression in cancer cells significantly reduced CD8+ T cell apoptosis from approximately 25% to 10% in co‐cultures with HCT116 cells and from 30% to 15% in co‐cultures with SW480 cells (Figure 7C,D). This protective effect on T cells was partially reversed by CDKN2A knockdown, which increased T cell apoptosis to approximately 20% in both co‐culture systems. These findings suggest that the SNHG26‐CDKN2A axis might induce T cell exhaustion rather than direct cytotoxicity, a mechanism that warrants further investigation. Beyond cell–cell interactions, we examined how the SNHG26‐CDKN2A axis influences cancer cell migration, a critical determinant of metastatic potential. Migration assays revealed that SNHG26 overexpression significantly suppressed the migratory capacity of both HCT116 and SW480 cells, reducing migration indices by approximately 50% (Figure 7E,F). This anti‐migratory effect was partially reversed by CDKN2A knockdown, suggesting a complex interplay between proliferation, cell death resistance and reduced metastatic potential. To elucidate the molecular basis for these immunomodulatory effects, we assessed the expression of key chemokines involved in T cell recruitment. Quantitative RT‐PCR analysis revealed that SNHG26 overexpression significantly downregulated CXCL9 and CXCL10 expression in both cell lines, with reductions of approximately 70% and 65%, respectively (Figure 7G,H). Concurrent CDKN2A knockdown partially restored chemokine expression, increasing CXCL9 and CXCL10 levels to approximately 60% and 55% of control levels, respectively.

## 4 Discussion

In this study, we have uncovered a novel regulatory axis in CRC involving the lncRNA SNHG26 and CDKN2A. Our findings reveal that SNHG26 functions as an oncogenic lncRNA that promotes CRC progression through post‐transcriptional destabilisation of CDKN2A mRNA, resulting in enhanced cell proliferation, resistance to cuproptosis and immune evasion. These discoveries provide significant insights into the molecular mechanisms underlying colorectal carcinogenesis and highlight potential therapeutic strategies.

lncRNAs have emerged as important regulators in various biological processes, functioning through diverse mechanisms to influence gene expression and cellular phenotypes [23, 24]. Our identification of SNHG26 as an oncogenic lncRNA in CRC adds to the growing repertoire of lncRNAs implicated in gastrointestinal malignancies. Notably, while several SNHG family members, including SNHG1, SNHG5 and SNHG16, have been previously associated with CRC [25, 26, 27], the role of SNHG26 has remained largely unexplored. Our comprehensive analysis demonstrates significant upregulation of SNHG26 in CRC tissues and its association with unfavourable clinical outcomes, establishing its clinical relevance.

The functional characterisation of SNHG26 through loss‐ and gain‐of‐function approaches revealed its profound impact on cellular proliferation, survival and clonogenic potential. These findings align with previous studies on other SNHG family members, which have similarly demonstrated pro‐proliferative and anti‐apoptotic functions in various cancer types [28, 29, 30]. However, our study extends beyond these canonical oncogenic properties by uncovering novel roles of SNHG26 in modulating sensitivity to cuproptosis and regulating immune interactions, dimensions that have not been previously explored for SNHG family lncRNAs. Notably, SNHG26 overexpression unexpectedly suppressed cell migration despite promoting proliferation and survival. This apparent paradox may be explained by the ‘go‐or‐grow’ dichotomy, where highly proliferative cells prioritise growth over motility, or by SNHG26 differentially regulating distinct molecular pathways controlling proliferation versus migration. Further investigation of SNHG26's effects on epithelial‐mesenchymal transition markers would help clarify this observation.

Mechanistically, we identified CDKN2A as a direct target of SNHG26 through RNA immunoprecipitation and RNA stability assays. While CDKN2A (p16INK4a) is classically recognised as a tumour suppressor that inhibits cyclin‐dependent kinases and induces cell cycle arrest [31], emerging evidence suggests context‐dependent oncogenic functions in certain cancer types [32]. Romagosa et al. demonstrated that p16INK4a overexpression correlates with high‐grade tumours and poor prognosis in various cancers, challenging the traditional tumour suppressor paradigm [32]. In CRC specifically, recent studies have reported CDKN2A upregulation and its association with enhanced metastatic potential through epithelial‐mesenchymal transition and resistance to chemotherapy [33, 34].

The apparent paradoxical function of CDKN2A as an oncogene in CRC may be explained by several plausible mechanisms. First, alternative splicing of CDKN2A could generate isoforms with altered functions, as different splice variants may have distinct cellular effects compared to the canonical p16INK4a protein. Second, post‐translational modifications, including phosphorylation and ubiquitination, could modulate p16INK4a stability and subcellular localisation, potentially converting it from a growth inhibitor to a growth promoter. Third, altered protein–protein interactions in the specific genetic context of CRC cells may redirect p16INK4a function, as suggested by the epithelial‐mesenchymal transition pathway involvement [33]. Fourth, the cellular context and co‐existing genetic alterations in CRC may reprogram p16INK4a from a cell cycle inhibitor to a promoter of cancer cell survival and drug resistance [34]. Our findings align with recent studies reporting CDKN2A upregulation in CRC tissues and its association with poor prognosis [22], supporting the notion that p16INK4a function is highly context‐dependent and can be reprogrammed in specific tumour environments.

The post‐transcriptional regulation of CDKN2A by SNHG26 represents a novel regulatory mechanism in CRC. Our discovery that SNHG26 directly interacts with CDKN2A mRNA to promote its degradation provides new insights into the complex regulatory network controlling CDKN2A expression. This mechanism is reminiscent of other lncRNA‐mRNA interactions, highlighting a conserved regulatory paradigm across different cancer types.

The molecular mechanism by which SNHG26 promotes CDKN2A mRNA degradation requires further investigation. Several hypotheses can be proposed based on established mRNA turnover pathways: SNHG26 may recruit RNA‐binding proteins that facilitate mRNA decay, serve as a scaffold for deadenylation complexes such as CCR4‐NOT, or expose CDKN2A mRNA to microRNA‐mediated degradation pathways. Additionally, SNHG26 could alter CDKN2A mRNA secondary structure, making it more susceptible to ribonuclease activity. Future RNA‐protein interaction studies could identify the specific factors mediating this regulatory mechanism.

One of the most significant findings of our study is the role of the SNHG26‐CDKN2A axis in modulating sensitivity to cuproptosis, a recently described form of programmed cell death triggered by copper‐dependent protein lipoylation [35]. Cuproptosis has emerged as a promising therapeutic strategy for cancer treatment, with several copper ionophores showing anticancer efficacy in preclinical models [36]. Our discovery that SNHG26 overexpression confers resistance to Cu + ELES‐induced cuproptosis through CDKN2A regulation provides, to our knowledge, the first evidence linking lncRNAs to this novel cell death pathway. This finding has important implications for understanding mechanisms of therapy resistance and developing strategies to enhance the efficacy of copper‐based therapeutics.

The dual role of the SNHG26‐CDKN2A axis in promoting cancer cell survival and facilitating immune evasion represents a potent mechanism for tumour progression. This multifaceted function is reminiscent of other oncogenic pathways, such as the PD‐L1/PD‐1 axis, which simultaneously inhibits T cell function and promotes cancer cell survival. Targeting such pathways has proven successful in cancer immunotherapy, suggesting that the SNHG26‐CDKN2A axis could represent a promising therapeutic target for CRC.

Despite these promising findings, our study has several limitations that warrant further investigation. The molecular details of how SNHG26 promotes CDKN2A mRNA degradation remain to be fully elucidated. Potential mechanisms include the recruitment of RNA‐binding proteins or ribonucleases to destabilise CDKN2A mRNA, or the modulation of microRNA binding sites. Additionally, while our in vitro experiments provide compelling evidence for the oncogenic role of the SNHG26‐CDKN2A axis, validation in animal models would strengthen the translational significance of our findings. Future xenograft studies examining tumour growth, cuproptosis sensitivity and immune cell infiltration in the context of SNHG26 modulation would help confirm the therapeutic potential of targeting this axis. In vivo models would also allow assessment of the systemic effects of SNHG26‐targeted therapies and their impact on the tumour microenvironment, particularly CD8+ T cell recruitment and function. Our prognostic analyses were based on a relatively small cohort of 23 paired samples, which limits the statistical power and generalisability of our survival findings. While the observed trends are consistent with larger datasets such as TCGA, validation in independent, larger cohorts is essential to confirm the prognostic significance of the SNHG26‐CDKN2A axis. Future multicenter studies with extended follow‐up periods and larger sample sizes would strengthen the clinical relevance of our findings and facilitate the development of reliable prognostic biomarkers.

In conclusion, our study identifies a novel regulatory axis in CRC involving the lncRNA SNHG26 and CDKN2A. This axis promotes cancer progression through multiple mechanisms, including enhanced cell proliferation, resistance to cuproptosis and immune evasion. These findings provide new insights into the complex molecular networks governing colorectal carcinogenesis and highlight potential avenues for therapeutic intervention. Targeting the SNHG26‐CDKN2A axis could represent a promising strategy to overcome therapy resistance and enhance the efficacy of existing treatments for CRC.

## Author Contributions

**Ziang Wan:** data curation (equal), formal analysis (equal), investigation (equal), software (equal), validation (equal), visualization (equal). **Shan Gao:** conceptualization (equal), data curation (equal), funding acquisition (equal), investigation (equal), methodology (equal), project administration (equal), resources (equal), supervision (equal), writing – original draft (equal).

## Conflicts of Interest

The authors declare no conflicts of interest.

## Data Availability

The data that support the findings of this study are available from the corresponding author upon reasonable request.
